# High-level expression of a novel thermostable and mannose-tolerant β-mannosidase from *Thermotoga thermarum* DSM 5069 in *Escherichia coli*

**DOI:** 10.1186/1472-6750-13-83

**Published:** 2013-10-08

**Authors:** Hao Shi, Yingjuan Huang, Yu Zhang, Wenqian Li, Xun Li, Fei Wang

**Affiliations:** 1 College of Chemical Engineering, Nanjing Forestry University, Nanjing 210037, China; 2 Jiangsu Key Lab of Biomass-Based Green Fuels and Chemicals, Nanjing 210037, China

**Keywords:** *Thermotoga thermarum*, β-mannosidase, Mannose-tolerant, Mannan, Thermostability, Mannooligosaccharides

## Abstract

**Background:**

Mannan is one of the primary polysaccharides in hemicellulose and is widely distributed in plants. β-Mannosidase is an important constituent of the mannan-degrading enzyme system and it plays an important role in many industrial applications, such as food, feed and pulp/paper industries as well as the production of second generation bio-fuel. Therefore, the mannose-tolerant β-mannosidase with high catalytic efficiency for bioconversion of mannan has a great potential in the fields as above.

**Results:**

A β-mannosidase gene (*Tth man5*) of 1,827 bp was cloned from the extremely thermophilic bacterium *Thermotoga thermarum* DSM 5069 that encodes a protein containing 608 amino acid residues, and was over-expressed in *Escherichia coli* BL21 (DE3). The results of phylogenetic analysis, amino acid alignment and biochemical properties indicate that the Tth Man5 is a novel β-mannosidase of glycoside hydrolase family 5. The optimal activity of the Tth Man5 β-mannosidase was obtained at pH 5.5 and 85°C and was stable over a pH range of 5.0 to 8.5 and exhibited 2 h half-life at 90°C. The kinetic parameters *K*_*m*_ and *V*_*max*_ values for *p*-nitrophenyl-β-D-mannopyranoside and 1,4-β-D-mannan were 4.36±0.5 mM and 227.27±1.59 μmol min^-1^ mg^-1^, 58.34±1.75 mg mL^-1^ and 285.71±10.86 μmol min^-1^ mg^-1^, respectively. The *k*_*cat*_/*K*_*m*_ values for *p*-nitrophenyl-β-D-mannopyranoside and 1,4-β-D-mannan were 441.35±0.04 mM^-1^ s^-1^ and 41.47±1.58 s^-1^ mg^-1^ mL, respectively. It displayed high tolerance to mannose, with a *K*_*i*_ value of approximately 900 mM.

**Conclusions:**

This work provides a novel and useful β-mannosidase with high mannose tolerance, thermostability and catalytic efficiency, and these characteristics constitute a powerful tool for improving the enzymatic conversion of mannan through synergetic action with other mannan-degrading enzymes.

## Background

Mannans are complex polysaccharides representing one of the major components of hemicellulose, consisting of four types: linear mannan, glucomannan, galactomannan, and galactoglucomanan [1]. Each of these polysaccharides has a β-1,4-linked backbone units including mannose or a combination of glucose and mannose residues, with the presence of α-1,6-linked side-chain substitutions [2]. It was reported that the hydrolysis of these polysaccharides requires several mannan-degrading enzymes, primarily including β-mannanase (EC 3.2.178), β-mannosidase (EC 3.2.1.25) and β-glucosidase (EC 3.2.1.21). Other enzymes such as α-galactosidase and mannan esterase are required to remove α-galactosyl and O-acetyl side-chain substituent. Among these enzymes, two types of mannan-degrading enzymes are necessary [3]. One endotype, β-mannanase, is responsible for the cleavage of β-1,4-linked mannose residues backbone randomly to generate mannooligosaccharides. Another exotype, β-mannosidase, hydrlyses the nonreducing end of mannooligosaccharides to release mannoses [2]. It is known that β-mannosidase is produced from plants, bacterial, fungi, invertebrates as well as some mammalian species [4,5]. Based on amino acid similarity and multi-domains, β-mannosidases have been mainly classified into glycoside hydrolase family (GHF) 1, 2 and 5 (http://www.cazy.org/). These β-mannosidases from different GHFs possess considerable industrial applications in many fields, such as food, feed and pulp/paper industries [6]. In addition, β-mannosidases have important role in saccharification of hemicellulose for fuel and other chemicals production. In human, lack of β-mannosidase can lead to β-mannosidosis [7,8].

During the last two decades, thermostable enzymes from thermophilic or hyperthermophilic microorganisms have become the hotspots of researches in many fields [9]. The amino acid sequences of β-mannosidases are abundantly available on the constantly updating databases. However, only a few β-mannosidases especially from hyperthermophile have been cloned, purified and characterized [3,5,10]. It was found that the known hyperthermophilic β-mannosidases from *Pyrococcus furiosus*, *Thermotoga maritima*, and *Thermotoga neapolitana* belonged to the GHF1, GHF2, and GHF2, respectively [11-13]. *Thermotoga thermarum*, isolated from continental solfataric springs at Lac Abbe (Djibouti, Africa), is an anaerobic hyperthermophilic bacteria that grows at 80°C [14]. And it has many glycoside hydrolase genes based on the genomic sequence (GenBank accession number: CP002351).

The biotechnology industry is essential in modern societies [15], which is reflected in the production of recombinant enzymes (including β-mannosidases) and their applications. In this study, we described the cloning, expression and functional characterizations of a novel recombinant β-mannosidase (Tth Man5) in *E. coli*.

## Results

### Amino acid sequence of Tth Man5 **β**-mannosidase

**Figure 1:**
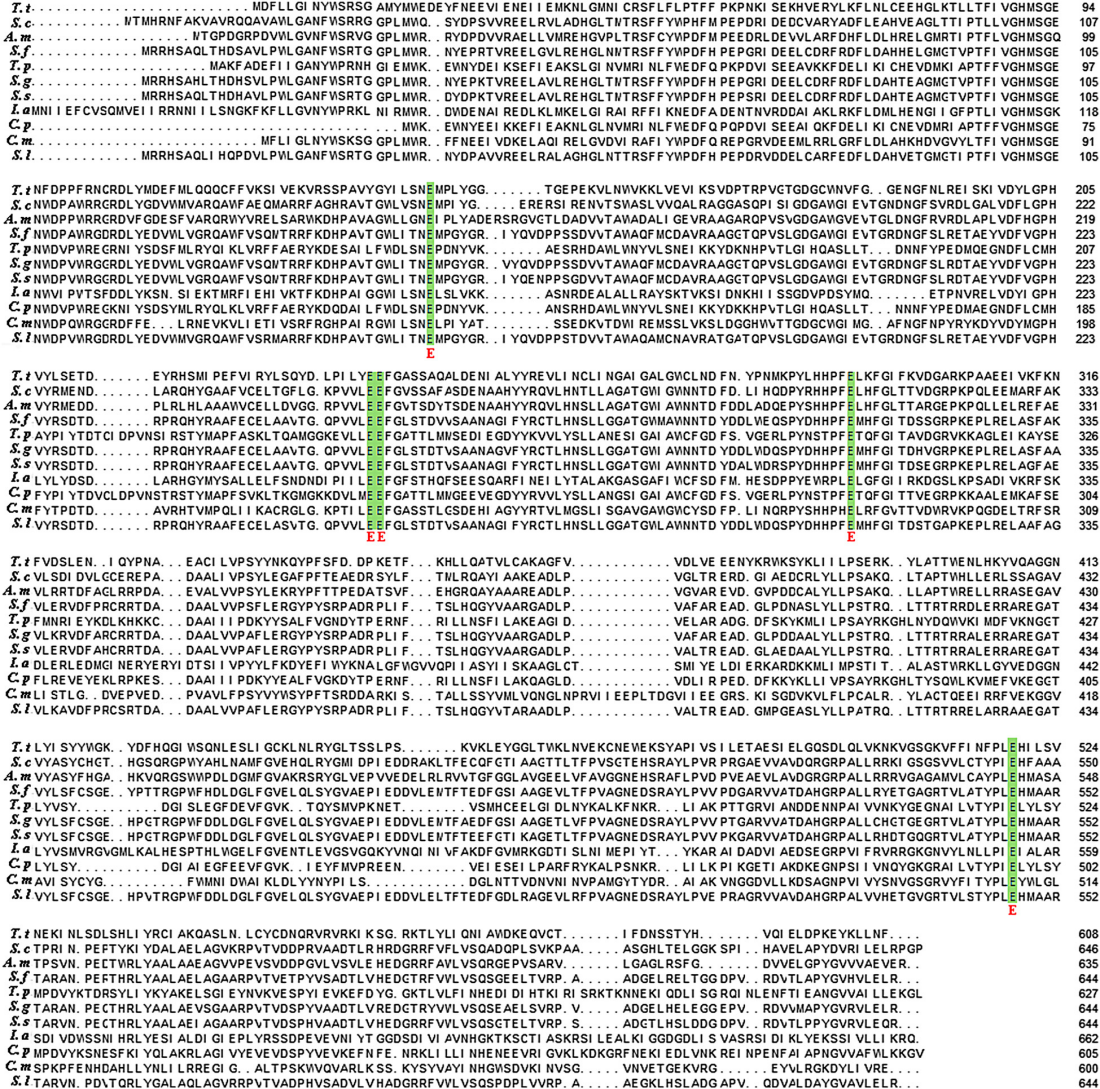
**Multi-alignment of Tth Man5 β-mannosidase with other GHF5 members.** Sequence alignment was performed by using Clustal X2.0. *Thermotoga thermarum* (*T. t*): GenBank No. AEH51033; *Sorangium cellulosum* (*S. c*): GeneBank No. YP_001611298; *Actinosynnema mirum* (*A. m*): GenBank No. YP_003101832; *Streptomyces flavogriseus* (*S. f*): GenBank No. YP_004925051; *Streptomyces globisporus* (*S. g*): GenBank No. ZP_11381673; *Streptomyces* sp. (*S. s*): GenBank No. ZP_09180515; *Ignisphaera aggregans* (*I. a*): GenBank No. YP_003859038; *Carboxydibrachium pacificum* (*C. p*): GenBank No. ZP_05092335; *Caldivirga maquilingensis* (*C. m*): GenBank No. YP_001540758; *Streptomyces lividans* (*S. l*): GenBank No. ZP_06533254.

The *Tth man5* gene isolated from the *T. thermarum* genome was 1,824 bp in length coding 608 amino acids and it was predicted as an endo-β-mannanase (Theth_0949) available at NCBI and CAZy sites (http://www.ncbi.nlm.nih.gov/, http://www.cazy.org/) (Lucas S etal, 2011). As shown in Figure 1, Tth Man5 displayed 33% identity to β-mannosidase from *Sorangium cellulosum* So ce56, 32% identity to putative β-mannosidase from *Actinosynnema mirum* DSM 43827 and 32% identity to the glycoside hydrolase from *Streptomyces flavogriseus* ATCC 33331. The results of alignments also revealed that Glu141, Glu237, Glu238, Glu292 and Glu591 were conserved amino acids among these GHF5 β-mannosidases. According to the CAZy database, two glutamic acids are the acid/base and the nucleophile, respectively. Against the similar catalytic domain of GHF5 endoglucanase (EXPDB No: 1TVP_A) from *Pseudoalteromonas haloplanktis*, it was presumed that active amino acids of Tth Man5 β-mannosidase were Glu141 and Glu238 [16].

### Over-expression and purification of Tth Man5 **β**-mannosidase

**Figure 2:**
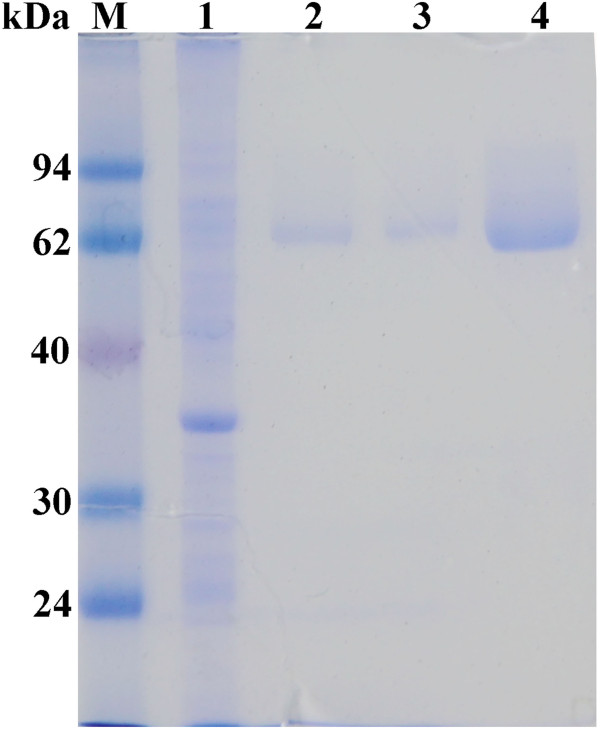
**SDS-PAGE analysis of recombinant Tth Man5 β-mannosidase in *E. coli *BL21 (DE3).** Lane M: protein marker, lane 1: cell-free extract of *E. coli* BL21 (DE3) harboring pET-20b plasmids, lane 2: the purified Tth Man5 β-mannosidase eluted with 0.2 M 1 mL imidazole buffer, lane 3,4: the purified Tth Man5 β-mannosidase eluted with 0.4 M 1 mL imidazole buffer (lane 3: first tube collection, lane 4: second tube collection).

When using native gene from *T. thermarum* for expression, the protein production was very difficult to detected (data not shown). Thus, in order to increase the expression level of Tth Man5 β-mannosidase in *Escherichia coli*, rare codons were replaced by optimal codons without change of amino acid sequence (data not shown). The mature protein without the signal peptide, allowing the insertion of a His_6_-tag at the C-terminus, was successfully expressed in *E. coli* BL21 (DE3), after induction with IPTG for 5 h at 37°C. The recombinant protein in the cell-free extract was purified by a heat treatment followed by a nickel affinity column (Table 1). Finally, the purified recombinant enzyme displayed a single band on SDS-PAGE with an estimated molecular weight (MW) of 70 kDa (Figure 2), which was consistent with the predicted MW of monomer (71, 725 Da). Size exclusion chromatography was also carried out using the AKTA*FPLC*™ system to compute the oligomerization state of the target protein. It was deduced that the native protein formed 7-mer in solution with a calculated MW 508,019 Da according to the calibration curve of the gel filtration column.

**Table 1:** **Purification of the recombinant Tth Man5** β**-mannosidase**

**Purification step**	**Total volume (mL)**	**Total activity (μmol min**^**-1**^**)**	**Total protein (mg)**	**Specific activity (μmol mg**^**-1**^**min**^**-1**^**)**	**Recovery (%)**	**Purification (fold)**
Crude extract^a^	10	2160	135	16	100	1
Heat treatment^b^	10	1922	31	62	89.0	3.9
Ni affinity chromatography^c^	1	1734	17	102	80.3	6.4

^a^The recombinant strain was grown in LB medium (200 ml) with 100 μg ampicillin/ml at 37°C to OD_600_ 0.4-0.5 and was incubated further with isopropyl-β-thiogalactopyranoside (IPTG) for 5 h. The cells were harvested by centrifugation at 10,000 g for 15 min at 4°C and resuspended in 10 ml imidazole buffer (10 mL of 5 mM imidazole, 0.5 mM NaCl, and 20 mM Tris–HCl buffer, pH 7.9), followed by sonication.

^b^The cell extracts after sonication were heat treated at 70°C for 30 min, and then cooled in an ice bath, centrifuged at 15,000 g for 20 min at 4°C and the supernatant was kept.

^c^The obtained supernatants were loaded on to an immobilized metal affinity column (Novagen, USA), and eluted with 0.4 M imidazole, 0.5 M NaCl, and 20 mM Tris–HCl buffer (pH 7.9).

### Biochemical characteristics of Tth Man5 **β**-mannosidase

The enzymatic properties of purified recombinant Tth Man5 β-mannosidase were determined and summarized in Tables 2, 3 and 4. Substrate specificity was assayed with different substrates and Tth Man5 β-mannosidase was found to be active to *p*-nitrophenyl-β-D-mannopyranoside (*p*NPM) and 1,4-β-D-mannan. However, no activity was detected towards konjaku powder, guar galactomannan and galactan (Table 2). These results indicated that the enzyme showed only exo-enzyme activity, which is not consistent with the prediction on its function at NCBI and CAZy.

**Table 2:** **Specific activity of Tth Man5** β**-mannosidase on various substrates**

**Substrate**	**Specific activity**
	**(μmol min**^**-1**^**mg**^**-1**^**)**
*p*-nitrophenyl-β-D-mannopyranoside	102.00±2.65
1,4-β-D-mannan	89.50±1.34
galactan	ND
konjaku powder	ND
guar galactomannan	ND

ND: not detected. Values shown were the mean of triplicate experiments, and the variation about the mean was below 5%.

**Table 3:** **Effects of cations and chemical reagents on purified Tth Man5** β**-mannosidase activity**

**Cations**^**a**^	**Residual activity (%)**
Control	100
Mg^2+^	114.85±5.50
Zn^2+^	25.63±2.20
Mn^2+^	129.61±2.88
Ba^2+^	107.96±3.84
Ca^2+^	103.01±2.33
Al^3+^	109.61±3.50
Cu^2+^	28.93±1.22
Co^2+^	191.94±7.27
Ni^2+^	81.07±0.69
Chemical reagents^b^	
EDTA	119.32±1.24
Tween 60	100.19±3.76
Tris	58.34±0.96
SDS	26.02±0.51

^a^Final concentration, the former value in the table was determined at 1 mM. ^b^Final concentration, the values in the table were determined at 1 mM, 0.05%, 0.05% and 0.1% for EDTA, Tween 60, Tris and SDS, respectively. Values shown were the means of triplicate experiments.

**Table 4:** **Characteristics of** β**-mannosidases from different sources for **^**a**^***p*NPM as substrate**

**Strain**	***V***_***max***_**(μmol min**^**-1**^**mg**^**-1**^**)**	***K***_***m***_**(mM)**	***k***_***cat***_**(s**^**-1**^**)**	***k***_***cat***_***/K***_***m***_**(mM**^**-1**^**s**^**-1**^**)**	***K***_***i***_**for mannose (mM)**	**Optimal Temp (°C)**	**Reference**
*Thermotoga thermarum*	227.27±1.59	4.36±0.05	1924.29±13.47	441.35±0.04	900	85	This work
*Thermotoga neapolitana*	36.9±2.5	3.1±2.5	^c^67.83	^c^24.23	^b^ND	90	11
*Thermotoga maritima*	50.5	0.49	^c^8.43	^c^17.22	ND	95	10
*Pyrococcus furiosus*	31.1	0.79	31.1	40	ND	105	12
*Thermoascus aurantiacus*	^c^3.66	1.1	6.1	5.5	ND	76	17
*Aspergillus niger*	^c^30	0.3	^c^67.5	^c^225	ND	70	16
*Thermobifida fusca*	5.96	0.18	^c^9.34	^c^51.89	5.5	53	22
*Aplysia kurodai*	3.75	0.1	^c^6.25	^c^62.5	ND	40	3

^a^*p*NPM: *p*-nitrophenyl-β-D-mannopyranoside.

^b^ND: not determined.

^c^Calculated by the data based on the reference.

**Figure 3:**
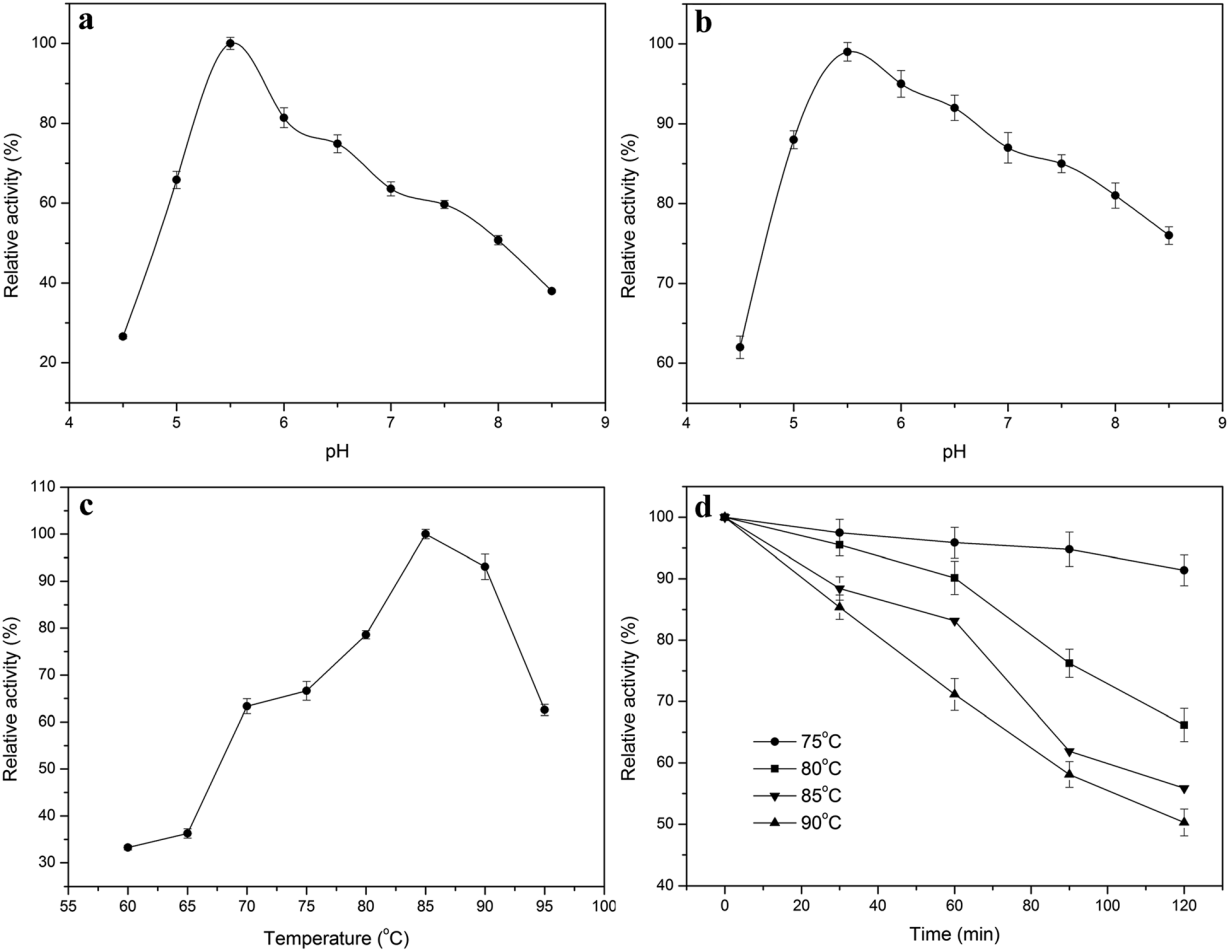
**Effects of pH and temperature on the activity and stability of the recombinant Tth Man5 β-mannosidase. a**. Optimal pH of the Tth Man5 β-mannosidase. **b**. pH stability of the Tth Man5 β-mannosidase. **c**. Effect of temperature on Tth Man5 β-mannosidase activity. **d**. The thermostability of the Tth Man5β-mannosidase. The residual activity was monitored, and the maximum activity was defined as 100% **(a, c)** or initial activity was defined as 100% **(b, d)**. Values shown were the mean of triplicate experiments, and the variation about the mean was below 5%.

The recombinant Tth Man5 β-mannosidase has a pH optimum of 5.5 as shown in Figure 3a. The enzyme was relatively stable at a pH range of 5.0 to 8.5 and most stable at pH 5.5 (Figure 3b). More than 70% of the initial enzyme activity remained at this range. The enzyme was most active at 85°C, and it retained approximately 50% of the maximum activity at 95°C (Figure 3c). The thermostability data showed that it remained above 56% of its initial activity after 2 h of pre-incubation at temperature ranging from 75°C to 90°C (Figure 3d).

**Figure 4:**
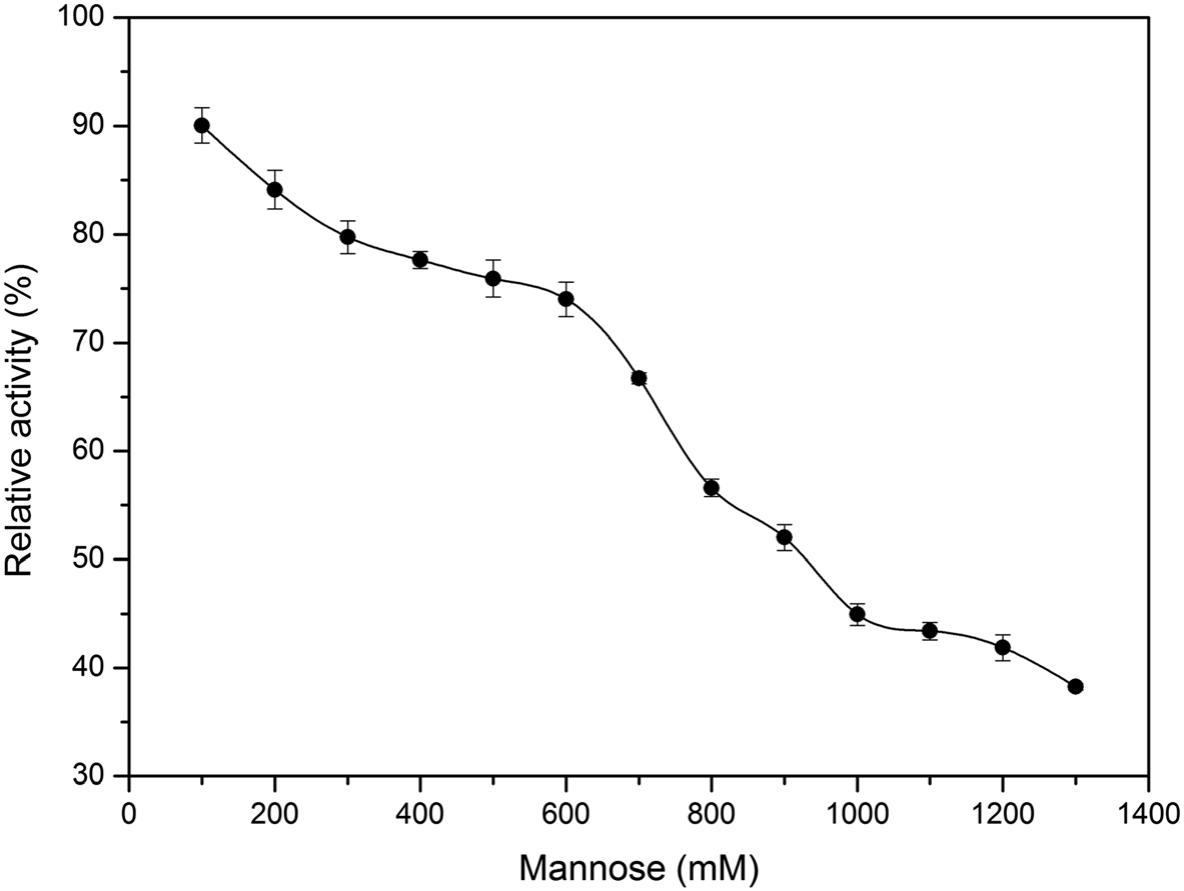
**Effect of mannose on Tth Man5 β-mannosidase activity using *p*-nitrophenyl-β-D-mannopyranoside as the substrate.** Values shown were the mean of three separate experiments, and the variations about the mean were all below 5%.

The effects of metal ions and chemical reagents on the enzyme activity are shown in Table 3. In various assays, the enzyme activity was apparently stimulated by 1 mM Mn^2+^, Co^2+^ and ethylene diamine tetraacetic acid (EDTA). However, the enzyme activity was apparently inhibited by 1 mM Cu^2+^ and Zn^2+^, 0.05% Tris and 0.1% SDS. Enzyme kinetic studies in the addition of *p*NPM and 1,4-β-D-mannan as the substrate at optimum temperature and pH allowed the determination of the Michaelis-Menten parameters (Table 4, data for 1,4-β-D-mannan was not summarized in this table). The enzyme had an obvious *K*_*m*_ of 4.36±0.5 mM , *V*_max_ of 227.27±1.59 μmol min^-1^ mg^-1^ and *k*_*cat*_/*K*_*m*_ of 441.35±0.04 mM^-1^ s^-1^ using *p*NPM as substrate, while *K*_*m*_ of 58.34±1.75 mg ml^-1^, *V*_max_ of 285.71±10.86 μmol min^-1^ mg^-1^ and *k*_*cat*_/*K*_*m*_ of 41.47±1.58 s^-1^ mg^-1^ mL for 1,4-β-D-mannan. The effect of mannose concentration on the Tth Man5 β-mannosidase activity was also investigated (shown in Figure 4). Though the enzyme activity was gradually decreased with the increase of mannose concentration, the enzyme could retain 50% of its initial activity at 900 mM of mannose concentration, indicating Tth Man5 β-mannosidase is a mannose-tolerant β-mannosidase with a *K*_*i*_ of 900 mM mannose.

### Hydrolysis of 1,4-**β**-D-mannan by the purified Tth Man5 **β**-mannosidase

**Figure 5:**
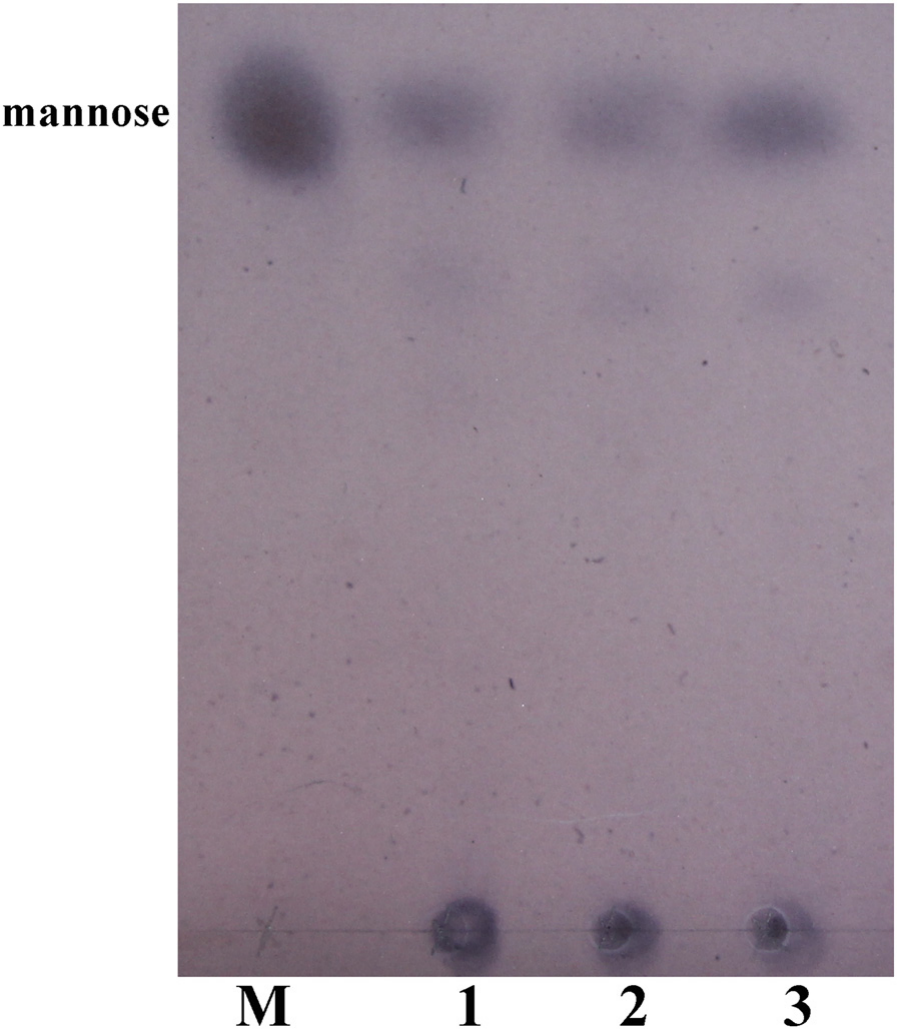
**Analysis of 1,4-β-D-mannan hydrolyzed by Tth Man5 β-mannosidase.** The products of the reaction were examined with TLC. M: mannose. Lane 1, 2, 3: 1,4-β-D-mannan (5%, wt/vol) incubated with 4 μg Tth Man5 b-mannosidase in 200 μL 50 mM imidazole-potassium buffer (pH 5.5) for 0.5 h, 1 h and 2 h, respectively.

Tth Man5 β-mannosidase was capable of degrading not only *p*NPM but also polymer mannan. The mode of action of Tth Man5 β-mannosidase was determined by analyzing the products of digestion of 1,4-β-D-mannan (Figure 5). The end products were almost the mannose after degradation for 0.5 h and mannose concentration increased with prolonging the hydrolysis time. The result confirmed that Tth Man5 β-mannosidase only displayed exo-enzyme activity.

### Phylogenetic analysis of Tth Man5 **β**-mannosidase

**Figure 6:**
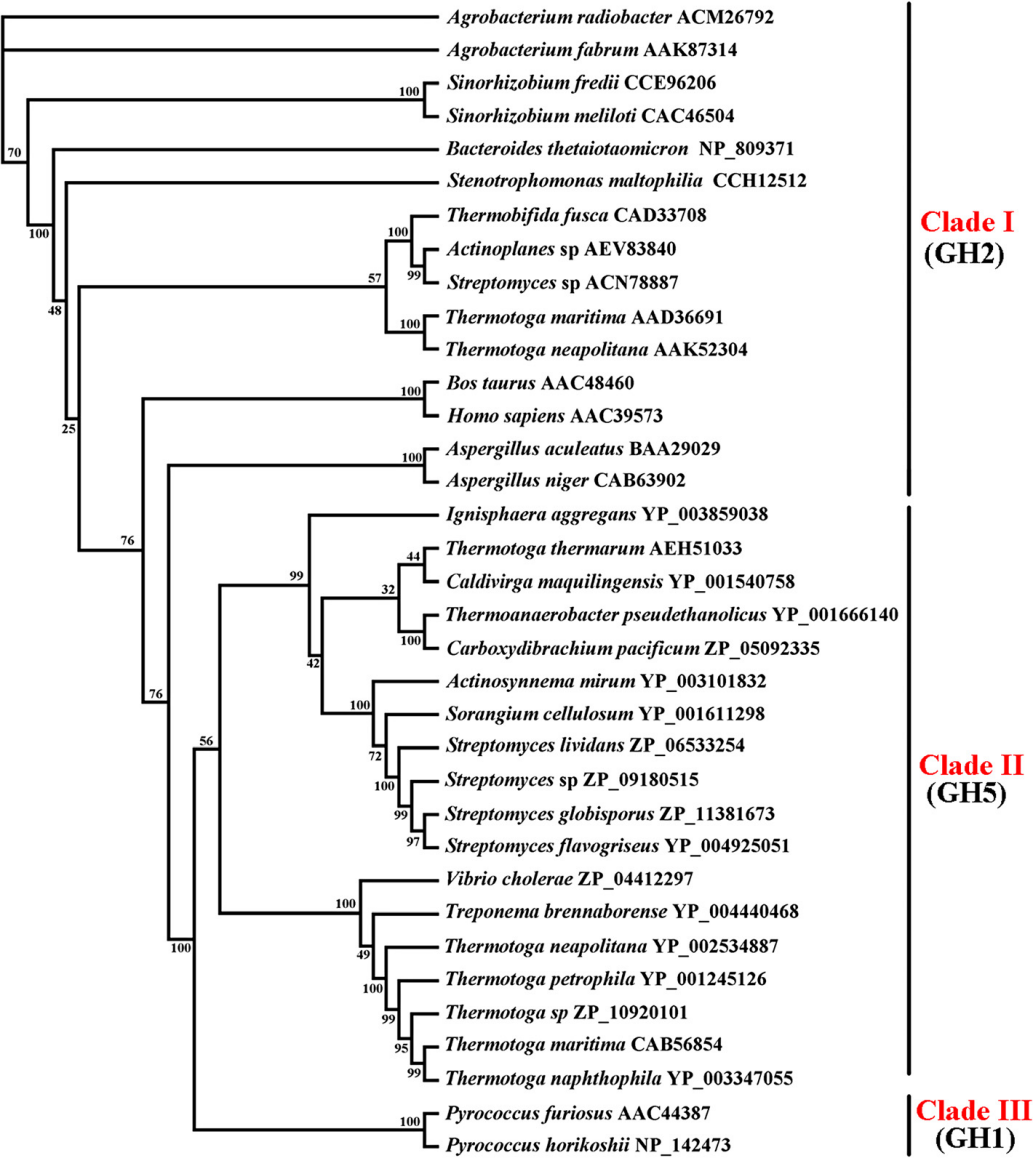
**Phylogentic tree resulted from analysis of β-mannosidases with 35 amino acid sequences using Maximum-Parsimony (MP) method.** Numbers on nodes correspond to percentage bootstrap values for 1000 replicates.

The phylogenetic trees generated from 35 candidate sequences were constructed to gain deeper insight into the evolutionary relationship among β-mannosidases, using the Neighbor-Joining (NJ) and Maximum-Parsimony (MP) methods. Both trees displayed almost the same topological structures (NJ tree was not shown). It revealed the presence of three well-supported clades and each clade consisting of a separated monophyletic group (Figure 6). Clade I, Clade II and Clade III consisted of the GHF2, GHF5 and GHF1 β-mannosidases, respectively. From the phylogenetic trees, it is obvious that there are two subclades in Clade II. Tth Man5 β-mannosidase from *T. thermarum* showed an apparently distant relationship with the GHF5 β-mannosidases from the same genus. Therefore, it was presumed that the biochemical properties of Tth Man5 β-mannosidase might differ from the same genus β-mannosidases. This was confirmed by the experiment results shown in Table 4.

## Discussion

β-Mannosidase is an important hydrolytic enzyme which attacks the non-reducing end of the β-linked mannooligosaccharides to release mannoses [17]. It plays a key role in the degradation pathway of complex oligosaccharide and glycoproteins [5]. To our knowledge, the hydrolytic end product by β-mannosidase, mannoses, are also fermentable sugars which can be bio-converted to bio-fuels and value-added chemicals [18]. The release of the genome in database provides us an effective way to investigate the uncharacterized enzymes, which may have great potential in industrial applications. For this study, a putative endo-β-mannanase gene (Theth_0949) from *T. thermarum* was cloned and was finally defined as a β-mannosidase through the biochemical characterization.

The *T. thermarum* β-mannosidase is distinct from the other glycosyl hydrolases from *T. thermarum.* The substrate specificity and amino acid sequence of the β-mannosidase are apparently different from those of the xylanase and β-xylosidase from *T. thermarum* previously reported [19,20]*.* Based on sequences similarity, the Tth Man5 β-mannosidase belongs to GHF5. It has a homologous relationship with those from *S. cellulosum* (33%), *A. mirum* (32%) and *S. flavogriseus* (32%) (Figure 1). Compared to the same genus *Thermotoga* β-mannosidase from GHF5 or GHF2, however, there is great difference according to phylogenetic analysis and they belong to different monophyletic groups (Figure 6). This suggests that Tth Man5 β-mannosidase may have some specific properties. Like other hemicellulases, the catalytic mechanism of β-mannosidase is also a double displacement lysozyme-like reaction, involving stabilization of an oxocarbonium ion by electrostatic interaction with two glutamate acid residues at the active site [2]. By the alignment with other GHF5 β-mannosidases and a GHF5 cellulase from *Pseudoalteromonas haloplanktis* (EXPDB No. 1TVP:A), the acid/base and the nucleophile are identified as Glu141 and Glu238 residues, respectively [16]. It is difficult to know more details about the functional amino acid residues as lack of a suitable template for homology modeling. Therefore, a crystalline structure analysis is needed to further investigate.

The Tth Man5 β-mannosidase from *T. thermarum* DSM 5069 exhibits the highest activity at temperature 85°C (Figure 3c). The temperature optima is higher than the β-mannosidases from *Homo sapiens*, *Aplysia kurodai*, *Bacillus licheniformis*, *Bacillus circulans*, *Aspergillus niger* and *Aspergillus awamori*, in which optimal temperature are ranged from 37°C to 70°C [3-5,17,21]. It is found that the β-mannosidases from the genus *Thermotoga* and *Pyrococcus furiosus* exhibit the maximum activity above 80°C [11-13]. However, pH optima for animals and fungi are near 5.0 while for bacteria occurs at neutral environment. As we know, the enzymes with high thermal stability are necessary in industrial progresses and thus it can minimize the enzyme consumption and enhance the catalytic efficiency. The Tth Man5 β-mannosidase residual activity retains approximately 91% after being incubated at 75°C for 2 h. Furthermore, the Tth Man5 β-mannosidase residual activity is still more than 50% even after being incubated at 90°C for 2 h. It shares similar thermostability at high temperature with the β-mannosidases from hyperthermophile. For instance, *T. neapolitana* β-mannosidase was stable at 90°C for 2 h and *T. maritima* β-mannosidase was stable at 80°C for 4 h [11,13]. The activity of β-mannosidase is significantly inhibited by Cu^2+^ and Zn^2+^ and this is similar to the β-mannosidase from *Homo sapiens* whose activity is thoroughly decreased the activity by the addition of 1.5 mM Cu^2+^ or Zn^2+^. However, the enzyme activity is greatly stimulated by Co^2+^ and Mn^2+^ (Table 3), very different from *Thermotoga maritima* β-mannosidase which enzyme activity is apparently inhibited by the both ions [11].

β-Mannosidase is known to be a key enzyme for converting mannooligosaccharides to mannoses, the end products, which are able to inhibit the enzymatic reaction to a certain extent. Therefore, β-mannosidases with high tolerance to mannoses are beneficial to conversion of mannooligosaccharides. However, little information about mannose-tolerant β-mannosidase is available in literatures. As shown in Figure 4, Tth Man5 β-mannosidase is insensitive to mannose (48.0% of inhibition at 900 mM) whereas the *K*_*i*_ for *Thermobifida fusca* β-mannosidase is only 5.5 mM [10], suggesting a high tolerance to mannose inhibition. Moreover, high enzyme activity is also demanded for β-mannosidase in enzymatic hydrolysis of mannooligosaccharides. The *V*_*max*_ value of Tth Man5 β-mannosidase for *p*NPM is 227.27±1.59 μmol min^-1^ mg^-1^, 7-fold higher than thermostable β-mannosidases from *T. neapolitana* and *P. furiosus*, 38-fold higher than *T. fusca* β-mannosidase and 62-fold than *Thermoascus aurantiacus* β-mannosidase [10,12,13,18]. The *k*_*cat*_/*K*_*m*_ of Tth Man5 β-mannosidase for *p*NPM is 441.35±0.04 mM^-1^ s^-1^, approximately 2-fold higher than *A. niger* β-mannosidase, 11-fold higher than the *P. furiosus* β-mannosidase, 18-fold higher than the *T. neapolitana* β-mannosidase and 80-fold higher than the *Thermoascus aurantiacus* β-mannosidase [12,13,17,18]. Generallly, it is noteworthy that Tth Man5 β-mannosidase has the highest *V*_*max*_ and *k*_*cat*_/*K*_*m*_ value (Table 4). Therefore, its mannose tolerance and high catalytic efficiency are desirable features for potential industrial applications.

Due to its theoretical and practical importance we investigated the hydrolysis of different substrates. When 1,4-β-D-mannan was degraded by Tth Man5 β-mannosidase, mannose was produced as a major product (Figure 5). It states that Tth Man5 β-mannosidase is a typical β-mannosidase and acts on polymer substrate with an exolytic manner. Such an exo-type action has been found in the β-mannosidase from a marine gastropod, *Aplysia kurodai*[3]. It is obvious that Tth Man5 β-mannosidase has a significant ability for the hydrolysis of 1,4-β-D-mannan (linear mannan by removing essentially all of the α-linked D-galactosyl residues from galactomannan with β-mannanase and α-galactosidase) with the *V*_*max*_ of 285.71±10.86 μmol min^-1^ mg^-1^. However, no activity is detected on konjaku powder and guar galactomannan which are not pretreated with any enzymes. The results indicate that Tth Man5 β-mannosidase can act on galactomannan cooperatively with β-mannanase and α-galactosidase to produce mannose efficiently but can not act with only Tth Man β-mannosidase. As Tth Man β-mannosidase can degrade 1,4-β-D-mannan with high efficiency, it is deduced that it also can efficiently hydrolyze mannooligosaccharides whose degree of polymerization is less than 1,4-β-D-mannan.

## Conclusions

In this study, a novel β-mannosidase (Tth Man5) from *T. thermarum* DSM 5069 was over-expressed in *E. coli* with some specific features. The results of phylogenetic analysis and biochemical properties showed that the Tth Man5 β-mannosidase was distant with the other genus *Thermotoga* β-mannosidases. As compared to other microorganisms, the Tth Man5 β-mannosidase possessed higher tolerance to mannose, higher catalytic efficiency and higher thermostability. Therefore, this study provides a novel and useful β-mannosidase with combined properties of high catalytic efficiency, mannose-tolerance and thermostability. This is easily envisioned that Tth Man5 β-mannosidase exhibits a great potential for enzymatic conversion of mannans.

## Methods

### Bacterial strains and growth conditions

*Thermotoga thermarum* DSM5069 was purchased from DSMZ; German Culture Collection of Microorganisms and Cell Cultures (Braunschweig, Germany). *Escherichia coli* Top10 (Novagen) was used for routine molecular cloning work and *E. coli* BL21 (DE3) (Novagen) as the host for expression the recombinant β-mannosidase. The vectors pET-20b and pET-28a were used for cloning and expression. Cells of recombinant strain (*E. coli* Top10 or *E. coli* BL21 (DE3)) harboring pET-20b-*Tth man5* and pET-28a-*Tth man5* were grown in Luria-Bertani (LB) medium with addition of ampicillin (100 μg ml^-1^) and kanamycin (50 μg ml^-1^), respectively. Isopropyl-β-D-thiogalactopyranoside (IPTG) was added with the concentration of 0.5 mM.

### Construction of plasmids and strains

DNA extraction was carried out as standard methods [22]. Restriction enzymes and DNA polymerase were purchased from Takara (Dalian, China) and used according to the manufacturer’s instructions. DNA was eluted from agarose gels with BIOMIGA Gel Extraction Kit (BIOMIGA, Shanghai). DNA sequencing was performed with ABI 3730 (Applied Biosystems). PCR amplifications were done using high-fidelity Ex-Taq DNA polymerase, and the resulting products purified by BIOMIGA PCR Purification Kit (BIOMIGA, Shanghai).

The coding sequence of *Tth man5* gene was amplified by PCR of genomic DNA using primer 1 and primer 2 as shown in Table 1. The synthesized codon optimized gene fragment was amplified using primer 3 and primer 4 as shown in Table 5. The primers 1 and 2 introduced the restrictions sites *Nco*I and *Xho*I at the 5’ and 3’ end, respectively. Primers 3 and 4 used *Nde*I and *Xho*I restrictions sites. PCR was performed as follows: 94°C, 5 min; 30 cycles of 94°C for 30 s, 55°C for 30 s and 72°C for 100 s; and 72°C, 10 min. The amplified DNA fragments were digested with the corresponding restriction endonucleases, and inserted into the corresponding sites in pET-28a and pET-20b (Novagen) to produce recombinant plasmids. The two plasmids encode a recombinant β-mannosidase bearing a C-terminal His_6_-tag, under the control of a T7 inducible promoter. The sequence of the inserts in pET-28a and pET-20b was confirmed by DNA sequencing.

**Table 5:** Nucleotide sequences of the primers used

**Primer**	**Nucleotide sequence**
1	5′-CATGCCATGGGCATGGATTTTCTTCTTGGCATCAATT-3′, Tm=62.7°C
2	5′-CCGCTCGAGAAAGTTCAGCAATTTGTACTCTTTG-3′, Tm=57.6°C
3	5′-GGAATTCCATATGGATTTCCTGCTGGGTATTAACTACT-3′, Tm=62.0
4	5′-CCGCTCGAGGAAGTTCAGCAGCTTATACTCTTTC-3′, Tm=56.7

### Expression and purification of recombinant β-mannosidase

*E. coli* BL21 (DE3) cells in 200 mL of LB with appropriate antibiotic selection harbouring recombinant plasmids were grown at 37°C and 200 rpm. When the OD_600_ reached 0.4 to 0.5, the expression of β-mannosidase was induced by the addition of 0.5 mM IPTG and the culture was incubated at 37°C and 200 rpm for 5 h. Cells were harvested by centrifugation at 4°C (10000 rpm, 5 min), washed twice with 20 mM Tris–HCl buffer (pH 8.0), and re-suspended in 5 mL of 5 mM imidazole, 0.5 mM NaCl, and 20 mM Tris–HCl buffer (pH 7.9). The cell extracts after sonication were heat treated at 70°C for 30 min, cooled in an ice bath, and then centrifuged (15000 g, 4°C, 20 min). The obtained supernatants were loaded on to an immobilized metal affinity column (2 mL) (Novagen, USA) with a flow rate 0.2 mL min^-1^. Finally, 1 mL fractions were collected by eluting with 0.4 M imidazole, 0.5 M NaCl, and 20 mM Tris–HCl buffer (pH 7.9). The fractions containing β-mannosidases were dialyzed overnight against storage buffer (20 mM Na-phosphate buffer, pH7.0, 50 mM NaCl, 10% glycerol) and then kept at -80°C until further use. The analysis of production, purity and molecular mass of the enzymes were determined by SDS-PAGE, using broad range molecular weight markers purchased from Thermo Fisher Scientific Inc. (12–94 kDa, MBI Fermemtas) as standards. The protein content was determined using Bradford reagent with albumin from bovine serum as standard. Oligomerization state of Tth Man β-mannosidase was determined by size exclusion chromatography on a AKTA*FPLC*™ (GE Healthcare Life Sciences) system with a Superdex 200 10/30 GL column as described by Zhang et al. [23].

### Enzyme assays

Substrate *p*NPM (Sigma, USA) was used for β-mannosidase activity analysis. Under standard assay condition, the purified enzyme (0.1 μg) was incubated with 10 μL of 20 mM substrate *p*NPM in 50 mM imidazole-potassium buffer (pH 5.5) for 10 min at 85°C. The total reaction volume was 0.2 mL. Subsequently, 600 μl of 1 M Na_2_CO_3_ was added to stop the reaction. The *p*-nitrophenol absorbance (*p*NP) was measured at 405 nm. One unit of enzyme activity was defined as the amount of enzyme necessary to liberate 1 μmol *p*NP per min under the assay conditions. All assays were performed in triplicate.

### Effect of temperature and pH on enzyme activity

The optimum pH for β-mannosidase was determined by incubation at various pH conditions (pH 4.5-8.5) at 85°C for 10 min in 50 mM imidazole-potassium buffer. The optimum temperature for the enzyme activity was determined by standard assay ranging from 60°C to 90°C in 50 mM imidazole-potassium buffer at pH 5.5. The results were expressed as relative activity to the value obtained at either optimum temperature or optimum pH. The maximum activity detected for pH optimum and temperature optimum were defined as 100%. PH stability assays were determined by measuring residual β-mannosidase activity after pre-incubation of enzymes in the pH rang of 4.5 to 8.5. Thermostability assays were determined by measuring residual β-mannosidase activity after pre-incubation of enzymes at 75°C, 80°C, 85°C, and 90°C for 30 min, 60 min, 90 min and 120 min. The activity of the enzyme without pre-incubation was defined as 100%.

### Effect of cations and chemical reagents

The effects of metal ions and chemical reagents on β-mannosidase activity of purified enzyme (0.1 μg) were determined. Mg^2+^, Zn^2+^, Mn^2+^, Ca^2+^, Al^3+^, Ni^2+^, Cu^2+^ and Co^2+^ were assayed at concentrations of 1 mM in the reaction mixture. The chemical reagents EDTA (1 mM), Tris (0.05%), Tween 60 (0.05%), and SDS (0.1%) in the 0.2 mL reaction mixture were assayed. The enzyme was incubated with each reagent for 1 h at 85°C before the addition of *p*NPM to start the enzyme reaction. The activity of the enzyme without the chemical reagents or metal cations was defined as 100%.

### Kinetic parameters and coefficient of mannose tolerance

Kinetic constant of β-mannosidase was determined by measuring the initial rates at various concentrations of *p*NPM (1 to 50 mM) under standard reaction conditions described as above. The influence of various mannose concentrations range from 0.1 M to 1.3 M on the β-mannosidase activity was investigated using *p*NPM as substrate. The *K*_*i*_ value of mannose was determined defined as amount of mannose required for inhibiting 50% of the β-mannosidase activity. All assays were performed in triplicate.

### Substrate specificity

The substrate specificity of the enzyme was determined by using following substrates, such as konjaku powder (Anhui, China), guar galactomannan (medium viscosity), 1,4-β-D-mannan and galactan (Megazyme International Ireland). The enzyme activities were assayed using the dinitrosalicylic acid (DNS) method [24]. The reaction mixture, containing 0.5% each substrate above and 0.1 μg enzyme in 0.2 mL 50 mM imidazole-potassium buffer (pH 5.5) reaction system, were incubated for 10 min at 85°C. The reaction was stopped by the addition of 0.3 mL DNS, followed by boiling for 5 minutes. The absorbance of the mixture was measured at 550 nm and converted to micromole of mannose by a mannose standard curve (data not shown). One unit of β-mannosidase activity was defined as the amount of enzyme releasing per μmol mannose per minute. Kinetic constant of β-mannosidase with each substrate was determined by measuring the initial rates at various concentrations from 1 mg mL^-1^ to 60 mg mL^-1^ under standard reaction conditions described as above. All assays were performed in triplicate.

### 1,4-β-D-mannan degradation

The 1,4-β-D-mannan was treated with purified Tth Man5 β-mannosidase, and the degradation was subjected to analysis on thin-layer chromatography (TLC). The reaction mixture (200 μL) contained 5% 1,4-β-D-mannan (wt/vol) and 4 μg of enzyme in 50 mM imidazole-potassium buffer (pH 5.5). The reaction was carried out for various times (0.5 h, 1 h and 2 h) at 80°C, and stopped in a water bath (4°C). After centrifuged for 10 min at 12,000 rpm, the supernatants of the reaction mixtures were applied on silica gel TLC plates (G, Qingdao). Sugars on the plates were separated with a solvent system consisting of *n*-butanol, acetic acid, and water (2:1:1, by vol/vol), and detected using the orcinol/concentrated sulfuric acid reagent [25].

### Bioinformatics analysis

A BLAST engine was used to search the amino acid sequences related to the β-mannosidase from *T. thermarum* and against the CAZy database. Clstal X2 was used for multiple sequence alignment [26]. Phylogenetic analysis was performed in Paup with the Neighbor-Joining (NJ) and Maximum-Parsimony (MP) methods [27].

### Amino acid sequence accession number

The GenBank accession number of β-mannosidase from *T. thermarum* DSM 5069 is AEH51033.

## Competing interests

The authors declare that they have no competing interests.

## Authors’ contributions

HS carried out the cloning, expression and drafted the manuscript. YH and YZ helped to purified and characterized the Tth Man5 β-mannosidase. WL and XL helped to analyze the data. FW directed the over-all study and revised the manuscript. All authors read and approved the final manuscript.
